# Tetra­aqua­(1,10-phenanthroline)nickel(II) 3,6-dicarboxy­bicyclo­[2.2.2]oct-7-ene-2,5-dicarboxyl­ate

**DOI:** 10.1107/S1600536809028190

**Published:** 2009-07-22

**Authors:** Yun-Yu Liu, Tian-Hui Wang, Zhi-Hao Wang, Yu-Jiang Zhuo, Xing-Qi Li

**Affiliations:** a Department of Chemistry, Northeast Normal University, Changchun 130024, People’s Republic of China; b Group of Chemistry, High School Attached to Northeast Normal University, Changchun 130024, People’s Republic of China; c Emergency Department, the First Clinical Hospital Affiliated to Jilin University, Changchun 130021, People’s Republic of China

## Abstract

In the title compound, [Ni(C_12_H_8_N_2_)(H_2_O)_4_](C_12_H_10_O_8_), the Ni^II^ ion is six-coordinated by two N atoms from one phenanthroline ligand and by the O atoms of four water mol­ecules in a distorted octa­hedral geometry. In the crystal, inter­molecular O—H⋯O hydrogen bonds form an extensive three-dimensional network, which consolidates the crystal packing.

## Related literature

For a related structure, see Liu *et al.* (2008 ▶).
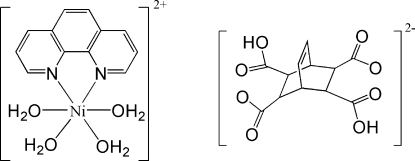


## Experimental

### 

#### Crystal data

[Ni(C_12_H_8_N_2_)(H_2_O)_4_](C_12_H_10_O_8_)
*M* *_r_* = 593.18
Monoclinic, 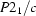

*a* = 7.446 (5) Å
*b* = 13.583 (6) Å
*c* = 22.982 (9) Å
β = 91.549 (7)°
*V* = 2323.5 (18) Å^3^
*Z* = 4
Mo *K*α radiation
μ = 0.91 mm^−1^
*T* = 293 K
0.30 × 0.28 × 0.17 mm

#### Data collection

Bruker APEX CCD area-detector diffractometer
Absorption correction: multi-scan (*SADABS*; Sheldrick 1996 ▶) *T*_min_ = 0.756, *T*_max_ = 0.855
14211 measured reflections
5636 independent reflections
4321 reflections with *I* > 2σ(*I*)
*R*_int_ = 0.032

#### Refinement

*R*[*F* ^2^ > 2σ(*F* ^2^)] = 0.041
*wR*(*F* ^2^) = 0.133
*S* = 1.10
5636 reflections
382 parameters
12 restraints
H atoms treated by a mixture of independent and constrained refinement
Δρ_max_ = 0.46 e Å^−3^
Δρ_min_ = −0.47 e Å^−3^

### 

Data collection: *SMART* (Bruker, 1998 ▶); cell refinement: *SAINT* (Bruker, 1998 ▶); data reduction: *SAINT*; program(s) used to solve structure: *SHELXS97* (Sheldrick, 2008 ▶); program(s) used to refine structure: *SHELXL97* (Sheldrick, 2008 ▶); molecular graphics: *SHELXTL-Plus* (Sheldrick, 2008 ▶); software used to prepare material for publication: *SHELXTL-Plus*.

## Supplementary Material

Crystal structure: contains datablocks global, I. DOI: 10.1107/S1600536809028190/cv2585sup1.cif

Structure factors: contains datablocks I. DOI: 10.1107/S1600536809028190/cv2585Isup2.hkl

Additional supplementary materials:  crystallographic information; 3D view; checkCIF report

## Figures and Tables

**Table 1:** Hydrogen-bond geometry (Å, °)

*D*—H⋯*A*	*D*—H	H⋯*A*	*D*⋯*A*	*D*—H⋯*A*
O7—H7⋯O3^i^	0.82	1.83	2.576 (3)	151
O1—H1⋯O6^ii^	0.82	1.88	2.670 (3)	163
O1*W*—H*W*11⋯O4	0.828 (16)	1.897 (18)	2.716 (3)	170 (3)
O1*W*—H*W*12⋯O5^iii^	0.859 (17)	1.828 (18)	2.682 (3)	173 (3)
O4*W*—H*W*41⋯O5^iv^	0.845 (18)	2.07 (3)	2.879 (3)	161 (4)
O4*W*—H*W*42⋯O3^v^	0.849 (18)	2.16 (2)	2.926 (3)	150 (4)
O3*W*—H*W*31⋯O3^vi^	0.863 (17)	1.958 (17)	2.814 (3)	171 (3)
O3*W*—H*W*32⋯O6^iii^	0.850 (17)	2.060 (19)	2.893 (3)	166 (4)
O2*W*—H*W*21⋯O3^v^	0.844 (18)	2.47 (2)	3.244 (4)	153 (4)
O2*W*—H*W*22⋯O2	0.853 (18)	2.01 (2)	2.806 (3)	154 (4)

Symmetry codes: (i) 
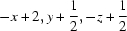
; (ii) 
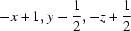
; (iii) 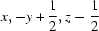; (iv) 
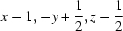
; (v) 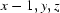; (vi) 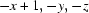.

**Fig. 1.**
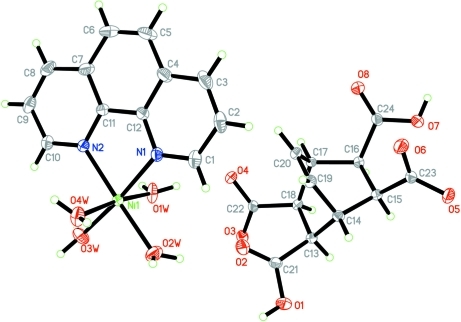
The structure of (I), showing the atomic numbering scheme. Displacement ellipsoids are drawn at the 30% probability level.

## supplementary crystallographic information

### Comment

Coordination polymers based on poly(carboxylic acids) have been investigated in the area of solid state and material science (Liu *et al.*, 2008). We selected bicyclo[2.2.2]oct-7-ene-2,3,5,6-tetracarboxylic acid (H_4_L) as a poly(carboxylic acid) ligand and phenanthroline (phen) as a secondary ligand, generating a complex, [Ni(phen)(H_2_O)_4_][(H_2_L)], which is reported here.

In the title compound (I) (Fig. 1), each Ni^II^ ion is six-coordinated by two N atoms from one phenanthroline molecule and by four O atoms from four water molecules in a distorted octahedral environment. In the crystal structure, ions are linked by O—H···O hydrogen bonds (Table 1).

### Experimental

A mixture of H_4_L (0.5 mmol), phen (0.5 mmol), NaOH (1 mmol) and NiCl_2_^.^2H_2_O (0.5 mmol) was suspended in 12 ml of deionized water and sealed in a 20-ml Teflon-lined autoclave. Upon heating at 100°C for one week, the autoclave was slowly cooled to room temperature. The crystals were collected, washed with deionized water and dried.

### Refinement

C-bound and hydroxy H atoms were geometrically positioned (C–H 0.93 Å, O–H 0.82 Å) and refined as riding,  with *U*_iso_(H)= 1.2-1.5 *U*_eq_ of the parent atom. The H atoms of the water molecules were located in a difference Fourier map and refined with an O—H distance restraint of 0.85±0.02 Å, and some of them were isotropically refined, while the rest of water' H-atoms were refined as riding with *U*_iso_(H)=1.5*U*_eq_(O).

### Crystal data

**Table:**

[Ni(C_12_H_8_N_2_)(H_2_O)_4_](C_12_H_10_O_8_)	*F*(000) = 1232
*M**_r_* = 593.18	*D*_x_ = 1.696 Mg m^−^^3^
Monoclinic, *P*2_1_/*c*	Mo *K*α radiation, λ = 0.71069 Å
Hall symbol: -P 2ybc	Cell parameters from 5636 reflections
*a* = 7.446 (5) Å	θ = 3.0–28.3°
*b* = 13.583 (6) Å	µ = 0.91 mm^−^^1^
*c* = 22.982 (9) Å	*T* = 293 K
β = 91.549 (7)°	Block, green
*V* = 2323.5 (18) Å^3^	0.30 × 0.28 × 0.17 mm
*Z* = 4	

### Data collection

**Table:**

Bruker APEX CCD area-detector diffractometer	5636 independent reflections
Radiation source: fine-focus sealed tube	4321 reflections with *I* > 2σ(*I*)
graphite	*R*_int_ = 0.032
φ and ω scans	θ_max_ = 28.3°, θ_min_ = 1.7°
Absorption correction: multi-scan (*SADABS*; Sheldrick 1996)	*h* = −9→8
*T*_min_ = 0.756, *T*_max_ = 0.855	*k* = −14→18
14211 measured reflections	*l* = −29→30

### Refinement

**Table:**

Refinement on *F*^2^	Primary atom site location: structure-invariant direct methods
Least-squares matrix: full	Secondary atom site location: difference Fourier map
*R*[*F*^2^ > 2σ(*F*^2^)] = 0.041	Hydrogen site location: inferred from neighbouring sites
*wR*(*F*^2^) = 0.133	H atoms treated by a mixture of independent and constrained refinement
*S* = 1.10	*w* = 1/[σ^2^(*F*_o_^2^) + (0.0737*P*)^2^ + 0.0226*P*] where *P* = (*F*_o_^2^ + 2*F*_c_^2^)/3
5636 reflections	(Δ/σ)_max_ = 0.001
382 parameters	Δρ_max_ = 0.46 e Å^−^^3^
12 restraints	Δρ_min_ = −0.47 e Å^−^^3^

### Special details

**Table:**

Geometry. All e.s.d.'s (except the e.s.d. in the dihedral angle between two l.s. planes) are estimated using the full covariance matrix. The cell e.s.d.'s are taken into account individually in the estimation of e.s.d.'s in distances, angles and torsion angles; correlations between e.s.d.'s in cell parameters are only used when they are defined by crystal symmetry. An approximate (isotropic) treatment of cell e.s.d.'s is used for estimating e.s.d.'s involving l.s. planes.
Refinement. Refinement of *F*^2^ against ALL reflections. The weighted *R*-factor *wR* and goodness of fit *S* are based on *F*^2^, conventional *R*-factors *R* are based on *F*, with *F* set to zero for negative *F*^2^. The threshold expression of *F*^2^ > σ(*F*^2^) is used only for calculating *R*-factors(gt) *etc*. and is not relevant to the choice of reflections for refinement. *R*-factors based on *F*^2^ are statistically about twice as large as those based on *F*, and *R*- factors based on ALL data will be even larger.

### Fractional atomic coordinates and isotropic or equivalent isotropic displacement parameters (Å^2^)

**Table:**

	*x*	*y*	*z*	*U*_iso_*/*U*_eq_	
Ni1	0.27952 (4)	0.16490 (2)	−0.000262 (12)	0.02453 (12)	
O3	0.8856 (3)	0.08862 (13)	0.10988 (7)	0.0376 (5)	
O8	0.8695 (3)	0.49129 (14)	0.26041 (8)	0.0426 (5)	
O5	0.7531 (3)	0.27133 (14)	0.41536 (7)	0.0373 (5)	
O6	0.5963 (3)	0.36870 (14)	0.35586 (8)	0.0399 (5)	
O7	0.9935 (3)	0.42968 (14)	0.34205 (8)	0.0401 (5)	
H7	1.0005	0.4878	0.3514	0.060*	
O4	0.7696 (3)	0.23715 (14)	0.09583 (8)	0.0441 (5)	
O1	0.6022 (3)	−0.02746 (14)	0.22071 (9)	0.0508 (6)	
H1	0.5256	−0.0589	0.2024	0.076*	
C14	0.6501 (3)	0.19162 (17)	0.27782 (10)	0.0236 (5)	
H14	0.5749	0.1518	0.3031	0.028*	
O4W	0.0076 (3)	0.13193 (18)	−0.00734 (9)	0.0413 (5)	
C18	0.8609 (3)	0.19220 (16)	0.19325 (9)	0.0232 (5)	
H18	0.9876	0.1783	0.2026	0.028*	
C23	0.7086 (3)	0.30189 (18)	0.36551 (10)	0.0261 (5)	
O2	0.5126 (3)	0.09191 (15)	0.16145 (9)	0.0502 (6)	
C5	0.2366 (5)	0.5506 (2)	0.02235 (17)	0.0571 (9)	
H5	0.2348	0.6111	0.0413	0.069*	
O3W	0.3341 (3)	0.06193 (16)	−0.06306 (9)	0.0455 (5)	
C17	0.8282 (3)	0.30067 (16)	0.20957 (9)	0.0215 (5)	
H17	0.8902	0.3447	0.1829	0.026*	
N2	0.2543 (3)	0.27843 (15)	−0.06039 (8)	0.0270 (4)	
C13	0.7483 (3)	0.12471 (17)	0.23318 (10)	0.0243 (5)	
H13	0.8303	0.0807	0.2547	0.029*	
C19	0.5369 (3)	0.26471 (17)	0.24367 (10)	0.0268 (5)	
H19	0.4132	0.2699	0.2472	0.032*	
N1	0.2510 (3)	0.28369 (16)	0.05628 (8)	0.0284 (5)	
O1W	0.5511 (3)	0.17925 (18)	0.00568 (9)	0.0436 (5)	
C16	0.9011 (3)	0.31475 (16)	0.27256 (10)	0.0232 (5)	
H16	1.0243	0.2893	0.2733	0.028*	
C12	0.2464 (3)	0.37093 (19)	0.02772 (11)	0.0292 (5)	
C20	0.6303 (3)	0.32107 (17)	0.20793 (10)	0.0268 (5)	
H20	0.5774	0.3688	0.1841	0.032*	
C22	0.8323 (4)	0.17204 (18)	0.12814 (10)	0.0270 (5)	
C15	0.7941 (3)	0.24788 (16)	0.31430 (9)	0.0236 (5)	
H15	0.8781	0.1991	0.3307	0.028*	
C4	0.2392 (4)	0.4628 (2)	0.05558 (13)	0.0421 (7)	
C24	0.9147 (4)	0.42251 (18)	0.29031 (10)	0.0286 (5)	
C21	0.6078 (4)	0.06232 (17)	0.20067 (11)	0.0291 (5)	
O2W	0.2891 (4)	0.05750 (18)	0.06329 (10)	0.0535 (6)	
C11	0.2481 (4)	0.36777 (18)	−0.03492 (11)	0.0295 (5)	
C9	0.2349 (4)	0.3574 (2)	−0.15295 (12)	0.0400 (7)	
H9	0.2291	0.3514	−0.1933	0.048*	
C2	0.2350 (4)	0.3740 (3)	0.14546 (12)	0.0435 (7)	
H2	0.2317	0.3726	0.1859	0.052*	
C1	0.2445 (4)	0.2857 (2)	0.11400 (11)	0.0377 (6)	
H1A	0.2464	0.2264	0.1343	0.045*	
C8	0.2324 (4)	0.4475 (2)	−0.12781 (13)	0.0440 (7)	
H8	0.2249	0.5037	−0.1509	0.053*	
C7	0.2409 (4)	0.4560 (2)	−0.06704 (13)	0.0410 (7)	
C3	0.2306 (4)	0.4607 (2)	0.11710 (14)	0.0491 (8)	
H3	0.2219	0.5192	0.1379	0.059*	
C10	0.2463 (4)	0.2735 (2)	−0.11797 (11)	0.0330 (6)	
H10	0.2484	0.2120	−0.1357	0.040*	
C6	0.2366 (5)	0.5477 (2)	−0.03657 (17)	0.0603 (10)	
H6	0.2338	0.6063	−0.0575	0.072*	
HW11	0.608 (4)	0.203 (2)	0.0338 (8)	0.035 (8)*	
HW12	0.618 (4)	0.190 (2)	−0.0236 (9)	0.063 (11)*	
HW41	−0.045 (6)	0.163 (3)	−0.0348 (13)	0.12 (2)*	
HW42	−0.063 (5)	0.128 (3)	0.0208 (12)	0.086 (14)*	
HW31	0.273 (4)	0.017 (2)	−0.0808 (14)	0.068 (12)*	
HW32	0.418 (4)	0.072 (3)	−0.0868 (14)	0.081*	
HW21	0.191 (3)	0.045 (3)	0.0794 (16)	0.081*	
HW22	0.370 (4)	0.051 (3)	0.0900 (14)	0.096 (16)*	

### Atomic displacement parameters (Å^2^)

**Table:**

	*U*^11^	*U*^22^	*U*^33^	*U*^12^	*U*^13^	*U*^23^
Ni1	0.02791 (19)	0.02203 (18)	0.02354 (18)	0.00082 (12)	−0.00149 (13)	0.00083 (12)
O3	0.0548 (13)	0.0316 (10)	0.0263 (9)	0.0051 (9)	−0.0030 (9)	−0.0066 (8)
O8	0.0632 (14)	0.0291 (10)	0.0349 (10)	−0.0053 (9)	−0.0107 (9)	0.0035 (8)
O5	0.0533 (13)	0.0376 (11)	0.0210 (8)	0.0078 (9)	0.0010 (8)	0.0027 (8)
O6	0.0540 (13)	0.0357 (11)	0.0300 (10)	0.0183 (9)	0.0041 (9)	0.0010 (8)
O7	0.0578 (13)	0.0266 (9)	0.0351 (10)	−0.0064 (9)	−0.0133 (9)	−0.0012 (8)
O4	0.0745 (16)	0.0287 (10)	0.0282 (9)	−0.0031 (9)	−0.0150 (9)	0.0067 (8)
O1	0.0688 (16)	0.0326 (11)	0.0503 (12)	−0.0225 (10)	−0.0133 (11)	0.0088 (9)
C14	0.0260 (12)	0.0218 (11)	0.0231 (11)	−0.0028 (9)	0.0042 (9)	0.0010 (9)
O4W	0.0308 (11)	0.0613 (14)	0.0319 (11)	−0.0050 (10)	0.0007 (9)	0.0032 (10)
C18	0.0272 (12)	0.0215 (11)	0.0210 (11)	0.0011 (9)	−0.0003 (9)	0.0019 (9)
C23	0.0334 (14)	0.0245 (12)	0.0206 (11)	−0.0027 (10)	0.0019 (10)	−0.0002 (9)
O2	0.0563 (14)	0.0408 (12)	0.0521 (13)	−0.0081 (10)	−0.0261 (11)	0.0006 (10)
C5	0.072 (2)	0.0250 (16)	0.074 (2)	0.0029 (14)	−0.0051 (19)	−0.0120 (16)
O3W	0.0501 (14)	0.0389 (12)	0.0476 (12)	−0.0010 (10)	0.0042 (10)	−0.0199 (10)
C17	0.0268 (12)	0.0186 (11)	0.0192 (10)	−0.0039 (9)	0.0000 (9)	0.0021 (9)
N2	0.0311 (11)	0.0262 (11)	0.0236 (10)	0.0009 (8)	−0.0011 (8)	0.0011 (8)
C13	0.0299 (13)	0.0205 (11)	0.0226 (11)	−0.0002 (9)	0.0005 (10)	0.0006 (9)
C19	0.0237 (12)	0.0268 (12)	0.0297 (12)	0.0013 (9)	−0.0028 (10)	−0.0004 (10)
N1	0.0308 (12)	0.0314 (12)	0.0229 (10)	0.0003 (9)	−0.0014 (8)	−0.0016 (9)
O1W	0.0281 (11)	0.0796 (17)	0.0229 (10)	−0.0082 (10)	−0.0016 (8)	−0.0010 (10)
C16	0.0246 (12)	0.0229 (12)	0.0219 (11)	−0.0008 (9)	−0.0010 (9)	−0.0004 (9)
C12	0.0309 (14)	0.0259 (12)	0.0307 (13)	0.0002 (10)	−0.0022 (10)	−0.0028 (11)
C20	0.0303 (13)	0.0246 (12)	0.0252 (12)	0.0038 (10)	−0.0053 (10)	0.0012 (9)
C22	0.0356 (14)	0.0270 (13)	0.0182 (11)	−0.0044 (10)	0.0003 (10)	−0.0001 (9)
C15	0.0276 (12)	0.0202 (11)	0.0228 (11)	0.0023 (9)	−0.0018 (9)	0.0005 (9)
C4	0.0464 (18)	0.0324 (15)	0.0469 (17)	0.0038 (12)	−0.0076 (13)	−0.0126 (13)
C24	0.0358 (14)	0.0268 (13)	0.0231 (12)	−0.0106 (10)	0.0023 (10)	−0.0009 (10)
C21	0.0374 (15)	0.0211 (12)	0.0289 (13)	−0.0029 (10)	0.0038 (11)	−0.0007 (10)
O2W	0.0576 (15)	0.0490 (13)	0.0531 (14)	−0.0126 (12)	−0.0113 (12)	0.0219 (11)
C11	0.0322 (14)	0.0238 (12)	0.0322 (13)	0.0004 (10)	−0.0035 (11)	0.0015 (10)
C9	0.0369 (16)	0.0559 (19)	0.0270 (13)	0.0007 (13)	−0.0006 (12)	0.0105 (13)
C2	0.0333 (16)	0.069 (2)	0.0281 (14)	0.0083 (14)	−0.0010 (11)	−0.0159 (14)
C1	0.0351 (15)	0.0514 (18)	0.0266 (13)	0.0016 (13)	0.0000 (11)	−0.0016 (12)
C8	0.0439 (17)	0.0444 (18)	0.0434 (16)	−0.0051 (13)	−0.0035 (13)	0.0230 (14)
C7	0.0465 (18)	0.0266 (14)	0.0497 (17)	−0.0016 (12)	−0.0031 (14)	0.0085 (12)
C3	0.0481 (19)	0.052 (2)	0.0463 (17)	0.0107 (14)	−0.0071 (14)	−0.0260 (16)
C10	0.0334 (14)	0.0390 (15)	0.0264 (12)	0.0019 (11)	−0.0015 (11)	0.0001 (11)
C6	0.082 (3)	0.0236 (15)	0.075 (2)	0.0015 (15)	−0.007 (2)	0.0080 (16)

### Geometric parameters (Å, °)

**Table:**

Ni1—O1W	2.033 (2)	N2—C10	1.325 (3)
Ni1—O3W	2.058 (2)	N2—C11	1.349 (3)
Ni1—O2W	2.064 (2)	C13—C21	1.526 (3)
Ni1—N2	2.076 (2)	C13—H13	0.9800
Ni1—O4W	2.076 (2)	C19—C20	1.332 (3)
Ni1—N1	2.086 (2)	C19—H19	0.9300
O3—C22	1.275 (3)	N1—C1	1.329 (3)
O8—C24	1.203 (3)	N1—C12	1.355 (3)
O5—C23	1.255 (3)	O1W—HW11	0.828 (16)
O6—C23	1.250 (3)	O1W—HW12	0.859 (17)
O7—C24	1.315 (3)	C16—C24	1.522 (3)
O7—H7	0.8200	C16—C15	1.556 (3)
O4—C22	1.238 (3)	C16—H16	0.9800
O1—C21	1.305 (3)	C12—C4	1.404 (4)
O1—H1	0.8200	C12—C11	1.441 (4)
C14—C19	1.509 (3)	C20—H20	0.9300
C14—C15	1.545 (3)	C15—H15	0.9800
C14—C13	1.566 (3)	C4—C3	1.417 (4)
C14—H14	0.9800	O2W—HW21	0.844 (18)
O4W—HW41	0.845 (18)	O2W—HW22	0.853 (18)
O4W—HW42	0.849 (18)	C11—C7	1.408 (4)
C18—C22	1.530 (3)	C9—C8	1.353 (4)
C18—C17	1.541 (3)	C9—C10	1.396 (4)
C18—C13	1.558 (3)	C9—H9	0.9300
C18—H18	0.9800	C2—C3	1.346 (5)
C23—C15	1.539 (3)	C2—C1	1.403 (4)
O2—C21	1.201 (3)	C2—H2	0.9300
C5—C6	1.355 (6)	C1—H1A	0.9300
C5—C4	1.416 (4)	C8—C7	1.401 (4)
C5—H5	0.9300	C8—H8	0.9300
O3W—HW31	0.863 (17)	C7—C6	1.430 (4)
O3W—HW32	0.850 (17)	C3—H3	0.9300
C17—C20	1.499 (3)	C10—H10	0.9300
C17—C16	1.544 (3)	C6—H6	0.9300
C17—H17	0.9800		
			
O1W—Ni1—O3W	84.11 (9)	Ni1—O1W—HW12	124 (2)
O1W—Ni1—O2W	90.27 (10)	HW11—O1W—HW12	105 (2)
O3W—Ni1—O2W	90.69 (11)	C24—C16—C17	112.92 (19)
O1W—Ni1—N2	92.61 (9)	C24—C16—C15	115.33 (19)
O3W—Ni1—N2	93.10 (9)	C17—C16—C15	109.46 (19)
O2W—Ni1—N2	175.45 (10)	C24—C16—H16	106.1
O1W—Ni1—O4W	173.00 (9)	C17—C16—H16	106.1
O3W—Ni1—O4W	90.51 (9)	C15—C16—H16	106.1
O2W—Ni1—O4W	85.31 (10)	N1—C12—C4	123.9 (2)
N2—Ni1—O4W	92.15 (9)	N1—C12—C11	117.2 (2)
O1W—Ni1—N1	90.11 (9)	C4—C12—C11	118.9 (2)
O3W—Ni1—N1	171.03 (9)	C19—C20—C17	114.0 (2)
O2W—Ni1—N1	96.20 (10)	C19—C20—H20	123.0
N2—Ni1—N1	80.28 (9)	C17—C20—H20	123.0
O4W—Ni1—N1	95.75 (9)	O4—C22—O3	123.6 (2)
C24—O7—H7	109.5	O4—C22—C18	119.9 (2)
C21—O1—H1	109.5	O3—C22—C18	116.4 (2)
C19—C14—C15	109.06 (19)	C23—C15—C14	110.8 (2)
C19—C14—C13	107.77 (18)	C23—C15—C16	114.98 (19)
C15—C14—C13	108.25 (19)	C14—C15—C16	108.26 (18)
C19—C14—H14	110.6	C23—C15—H15	107.5
C15—C14—H14	110.6	C14—C15—H15	107.5
C13—C14—H14	110.6	C16—C15—H15	107.5
Ni1—O4W—HW41	112 (3)	C12—C4—C5	120.2 (3)
Ni1—O4W—HW42	125 (3)	C12—C4—C3	116.1 (3)
HW41—O4W—HW42	108 (3)	C5—C4—C3	123.7 (3)
C22—C18—C17	112.97 (19)	O8—C24—O7	124.8 (2)
C22—C18—C13	114.05 (19)	O8—C24—C16	125.3 (2)
C17—C18—C13	109.14 (18)	O7—C24—C16	109.8 (2)
C22—C18—H18	106.7	O2—C21—O1	123.7 (2)
C17—C18—H18	106.7	O2—C21—C13	124.4 (2)
C13—C18—H18	106.7	O1—C21—C13	112.0 (2)
O6—C23—O5	124.1 (2)	Ni1—O2W—HW21	116 (3)
O6—C23—C15	119.9 (2)	Ni1—O2W—HW22	127 (3)
O5—C23—C15	115.8 (2)	HW21—O2W—HW22	105 (2)
C6—C5—C4	121.0 (3)	N2—C11—C7	122.7 (2)
C6—C5—H5	119.5	N2—C11—C12	117.5 (2)
C4—C5—H5	119.5	C7—C11—C12	119.8 (2)
Ni1—O3W—HW31	135 (2)	C8—C9—C10	119.6 (3)
Ni1—O3W—HW32	120 (2)	C8—C9—H9	120.2
HW31—O3W—HW32	102 (2)	C10—C9—H9	120.2
C20—C17—C18	109.41 (19)	C3—C2—C1	120.0 (3)
C20—C17—C16	108.71 (18)	C3—C2—H2	120.0
C18—C17—C16	107.02 (18)	C1—C2—H2	120.0
C20—C17—H17	110.5	N1—C1—C2	122.4 (3)
C18—C17—H17	110.5	N1—C1—H1A	118.8
C16—C17—H17	110.5	C2—C1—H1A	118.8
C10—N2—C11	118.6 (2)	C9—C8—C7	120.0 (3)
C10—N2—Ni1	128.91 (18)	C9—C8—H8	120.0
C11—N2—Ni1	112.52 (16)	C7—C8—H8	120.0
C21—C13—C18	114.28 (19)	C8—C7—C11	116.9 (3)
C21—C13—C14	108.5 (2)	C8—C7—C6	124.0 (3)
C18—C13—C14	108.22 (18)	C11—C7—C6	119.1 (3)
C21—C13—H13	108.6	C2—C3—C4	119.9 (3)
C18—C13—H13	108.6	C2—C3—H3	120.0
C14—C13—H13	108.6	C4—C3—H3	120.0
C20—C19—C14	113.9 (2)	N2—C10—C9	122.3 (3)
C20—C19—H19	123.0	N2—C10—H10	118.9
C14—C19—H19	123.0	C9—C10—H10	118.9
C1—N1—C12	117.7 (2)	C5—C6—C7	121.0 (3)
C1—N1—Ni1	130.1 (2)	C5—C6—H6	119.5
C12—N1—Ni1	112.11 (16)	C7—C6—H6	119.5
Ni1—O1W—HW11	125.5 (19)		
			
C22—C18—C17—C20	74.5 (2)	O6—C23—C15—C16	60.0 (3)
C13—C18—C17—C20	−53.5 (2)	O5—C23—C15—C16	−123.0 (2)
C22—C18—C17—C16	−167.88 (19)	C19—C14—C15—C23	72.3 (2)
C13—C18—C17—C16	64.1 (2)	C13—C14—C15—C23	−170.72 (18)
O1W—Ni1—N2—C10	94.5 (2)	C19—C14—C15—C16	−54.7 (2)
O3W—Ni1—N2—C10	10.3 (2)	C13—C14—C15—C16	62.3 (2)
O2W—Ni1—N2—C10	−136.3 (11)	C24—C16—C15—C23	3.4 (3)
O4W—Ni1—N2—C10	−80.4 (2)	C17—C16—C15—C23	−125.2 (2)
N1—Ni1—N2—C10	−175.8 (2)	C24—C16—C15—C14	127.9 (2)
O1W—Ni1—N2—C11	−84.57 (18)	C17—C16—C15—C14	−0.7 (3)
O3W—Ni1—N2—C11	−168.81 (18)	N1—C12—C4—C5	179.4 (3)
O2W—Ni1—N2—C11	44.7 (12)	C11—C12—C4—C5	−1.0 (4)
O4W—Ni1—N2—C11	100.56 (18)	N1—C12—C4—C3	−2.1 (4)
N1—Ni1—N2—C11	5.09 (17)	C11—C12—C4—C3	177.5 (3)
C22—C18—C13—C21	−9.4 (3)	C6—C5—C4—C12	1.6 (6)
C17—C18—C13—C21	118.0 (2)	C6—C5—C4—C3	−176.8 (4)
C22—C18—C13—C14	−130.4 (2)	C17—C16—C24—O8	2.3 (4)
C17—C18—C13—C14	−3.0 (3)	C15—C16—C24—O8	−124.5 (3)
C19—C14—C13—C21	−67.1 (2)	C17—C16—C24—O7	−173.9 (2)
C15—C14—C13—C21	175.12 (19)	C15—C16—C24—O7	59.2 (3)
C19—C14—C13—C18	57.4 (2)	C18—C13—C21—O2	−42.5 (4)
C15—C14—C13—C18	−60.4 (2)	C14—C13—C21—O2	78.3 (3)
C15—C14—C19—C20	58.8 (3)	C18—C13—C21—O1	137.8 (2)
C13—C14—C19—C20	−58.5 (3)	C14—C13—C21—O1	−101.3 (2)
O1W—Ni1—N1—C1	−89.4 (2)	C10—N2—C11—C7	−2.6 (4)
O3W—Ni1—N1—C1	−139.2 (5)	Ni1—N2—C11—C7	176.5 (2)
O2W—Ni1—N1—C1	0.8 (3)	C10—N2—C11—C12	176.5 (2)
N2—Ni1—N1—C1	177.9 (3)	Ni1—N2—C11—C12	−4.4 (3)
O4W—Ni1—N1—C1	86.7 (2)	N1—C12—C11—N2	−0.1 (4)
O1W—Ni1—N1—C12	87.52 (18)	C4—C12—C11—N2	−179.7 (2)
O3W—Ni1—N1—C12	37.8 (6)	N1—C12—C11—C7	179.1 (2)
O2W—Ni1—N1—C12	177.81 (18)	C4—C12—C11—C7	−0.6 (4)
N2—Ni1—N1—C12	−5.11 (16)	C12—N1—C1—C2	−0.6 (4)
O4W—Ni1—N1—C12	−96.31 (18)	Ni1—N1—C1—C2	176.2 (2)
C20—C17—C16—C24	−74.2 (2)	C3—C2—C1—N1	0.6 (4)
C18—C17—C16—C24	167.72 (19)	C10—C9—C8—C7	−0.1 (5)
C20—C17—C16—C15	55.7 (2)	C9—C8—C7—C11	−1.3 (5)
C18—C17—C16—C15	−62.3 (2)	C9—C8—C7—C6	−179.1 (3)
C1—N1—C12—C4	1.4 (4)	N2—C11—C7—C8	2.7 (4)
Ni1—N1—C12—C4	−175.9 (2)	C12—C11—C7—C8	−176.4 (3)
C1—N1—C12—C11	−178.2 (2)	N2—C11—C7—C6	−179.3 (3)
Ni1—N1—C12—C11	4.4 (3)	C12—C11—C7—C6	1.6 (4)
C14—C19—C20—C17	−0.5 (3)	C1—C2—C3—C4	−1.3 (5)
C18—C17—C20—C19	58.8 (3)	C12—C4—C3—C2	2.0 (5)
C16—C17—C20—C19	−57.7 (3)	C5—C4—C3—C2	−179.6 (3)
C17—C18—C22—O4	−7.5 (3)	C11—N2—C10—C9	1.1 (4)
C13—C18—C22—O4	117.9 (3)	Ni1—N2—C10—C9	−177.9 (2)
C17—C18—C22—O3	168.6 (2)	C8—C9—C10—N2	0.2 (4)
C13—C18—C22—O3	−66.0 (3)	C4—C5—C6—C7	−0.6 (6)
O6—C23—C15—C14	−63.2 (3)	C8—C7—C6—C5	176.8 (4)
O5—C23—C15—C14	113.8 (2)	C11—C7—C6—C5	−1.0 (6)

### Hydrogen-bond geometry (Å, °)

**Table:**

*D*—H···*A*	*D*—H	H···*A*	*D*···*A*	*D*—H···*A*
O7—H7···O3^i^	0.82	1.83	2.576 (3)	151
O1—H1···O6^ii^	0.82	1.88	2.670 (3)	163
O1W—HW11···O4	0.83 (2)	1.90 (2)	2.716 (3)	170 (3)
O1W—HW12···O5^iii^	0.86 (2)	1.83 (2)	2.682 (3)	173 (3)
O4W—HW41···O5^iv^	0.85 (2)	2.07 (3)	2.879 (3)	161 (4)
O4W—HW42···O3^v^	0.85 (2)	2.16 (2)	2.926 (3)	150 (4)
O3W—HW31···O3^vi^	0.86 (2)	1.96 (2)	2.814 (3)	171 (3)
O3W—HW32···O6^iii^	0.85 (2)	2.06 (2)	2.893 (3)	166 (4)
O2W—HW21···O3^v^	0.84 (2)	2.47 (2)	3.244 (4)	153 (4)
O2W—HW22···O2	0.85 (2)	2.01 (2)	2.806 (3)	154 (4)

Symmetry codes: (i) −*x*+2, *y*+1/2, −*z*+1/2; (ii) −*x*+1, *y*−1/2, −*z*+1/2; (iii) *x*, −*y*+1/2, *z*−1/2; (iv) *x*−1, −*y*+1/2, *z*−1/2; (v) *x*−1, *y*, *z*; (vi) −*x*+1, −*y*, −*z*.
